# Spatial distribution data of cultural sites from the Paleolithic to Bronze Age in Xinjiang, China

**DOI:** 10.1038/s41597-022-01306-5

**Published:** 2022-05-13

**Authors:** Bo Tan, Hongwei Wang, Xiaoqin Wang, Suyan Yi, Jing Zhou, Chen Ma, Xinyan Dai

**Affiliations:** 1 Key Laboratory of Oasis Ecology, Xinjiang University, Urumqi, 830046 China; 2 College of Geography and Remote sensing Sciences, Xinjiang University, Urumqi, 830046 China

**Keywords:** Geography, Archaeology

## Abstract

The published map recording cultural sites in Xinjiang shows that there is a lack of data collection on the distribution of sites in the area, and no relevant data sets have been released. Existing written materials indicate that there are more cultural sites in this area. For this reason, we have collected and sorted out information. Our cultural site database provides the geographic location and corresponding geographic environment of each site in Xinjiang from the Paleolithic to the Bronze Age. The data record the human development and settlement process, settlement environment landscape characteristics, scale, type, quantity, and spatial distribution in Xinjiang in prehistoric China. These data not only are the basis for further understanding the spatial distribution of prehistoric humans in Xinjiang, but also provide references for understanding prehistoric human behavior and prehistoric man-land relationship, and the exchange of eastern and western civilizations. It is of great significance to modern social planning, site protection, and resource utilization.

## Abstract (summary)

**Table:** 

Measurement(s)	spatial distribution data of cultural sites
Technology Type(s)	Geographic Information System • digital curation
Independent variable(s)	geographic location • age • geographical landscape type
Sample Characteristic - Environment	prehistoric human sites
Sample Characteristic - Location	China

## Background & Summary

Human relics are the spatial relics of early human activities. They carry and record information about early human adaptation and the transformation of the environment. Interpreting and revealing the environmental information of settlement distribution can provide conditions for further understanding of the interaction between humans and the environment during this period. Xinjiang is located at the intersection of China and middle Asia, which is necessary for cultural exchanges between the East and the West^1^. Since the Holocene, the process of the dry, wet, cold and hot climate in Xinjiang has prompted corresponding changes in the natural environment and human culture^2–4^. A rich cultural type was born through repeated adaptation, selection, and exchanges^1^, making Xinjiang ideal conditions for conducting environmental, archaeological research. Archaeological excavations have shown that early human activities in Xinjiang can be traced back to the late Paleolithic period^5^, in the Gouxi platform of the Jiaohe Gucheng in Turpan, the Tongtian Cave of Jimunai, and Buxaer. A large number of stone-making tools have been discovered at the Luotuo Stone Paleolithic site. In the vast areas in the north and south of the Tianshan Mountains in Xinjiang, there are a great many Mesolithic fine stone and Neolithic remains. Fine stone tools, stone chips, stone core stone tools, grinding discs, grinding rods, stone balls, and grinding jade axes have been unearthed. There is also a small amount of painted pottery and embossed and carved pottery fragments on the jade adze. Around 4500a.Bp-2250a.Bp (Before Present), the oasis in Xinjiang entered the Bronze Age and the Early Iron Age successively^1,6–9^, and a large number of settlement sites remain.

In the existing research, there are two research papers extracting information such as the location of cultural sites from the Paleolithic to the Bronze Age in Xinjiang. The number of sites extracted is 41 records in the Stone Age and 194 records in the Bronze Age^10^, the other is the 35 and 42 records of the Stone Age^11^, there is a big difference in terms of quantity, and a big difference in terms of spatial positioning, and the results of data collection are uneven. Judging from the existing text records (National Cultural Heritage Administration, 2012; Shuitao, 2001; Guowu, 2012; Chinese Archaeological Society, 1984–2018)^12–16^, The existing data collection of cultural sites in Xinjiang has the problems of insufficient quantity and low spatial location accuracy. At the same time, the data results cannot be obtained publicly, so there are some obstacles to further data information mining. Therefore, we collected and recorded location information and geographic environment information for human cultural sites from the Paleolithic to the Early Bronze Age in Xinjiang. Referring to the role of the Amazon site database, these data can provide a data basis for archaeologists, landscape scientists, anthropologists, geographers, historians, heritage experts, palynologists and ecologists to study the human settlement process and behavior, the development and exchange of human civilization, resource use, plant domestication, landscape transformation and the evolution of human relations in Xinjiang^17^. These data have a positive effect on enriching and exploring the evolution of civilizations in Xinjiang, China, and Central Asia. At the same time, the prediction research based on the analysis of the status quo of the site distribution also has a specific reference value for the protection and excavation of the site.

## Methods

**Fig. 1:**
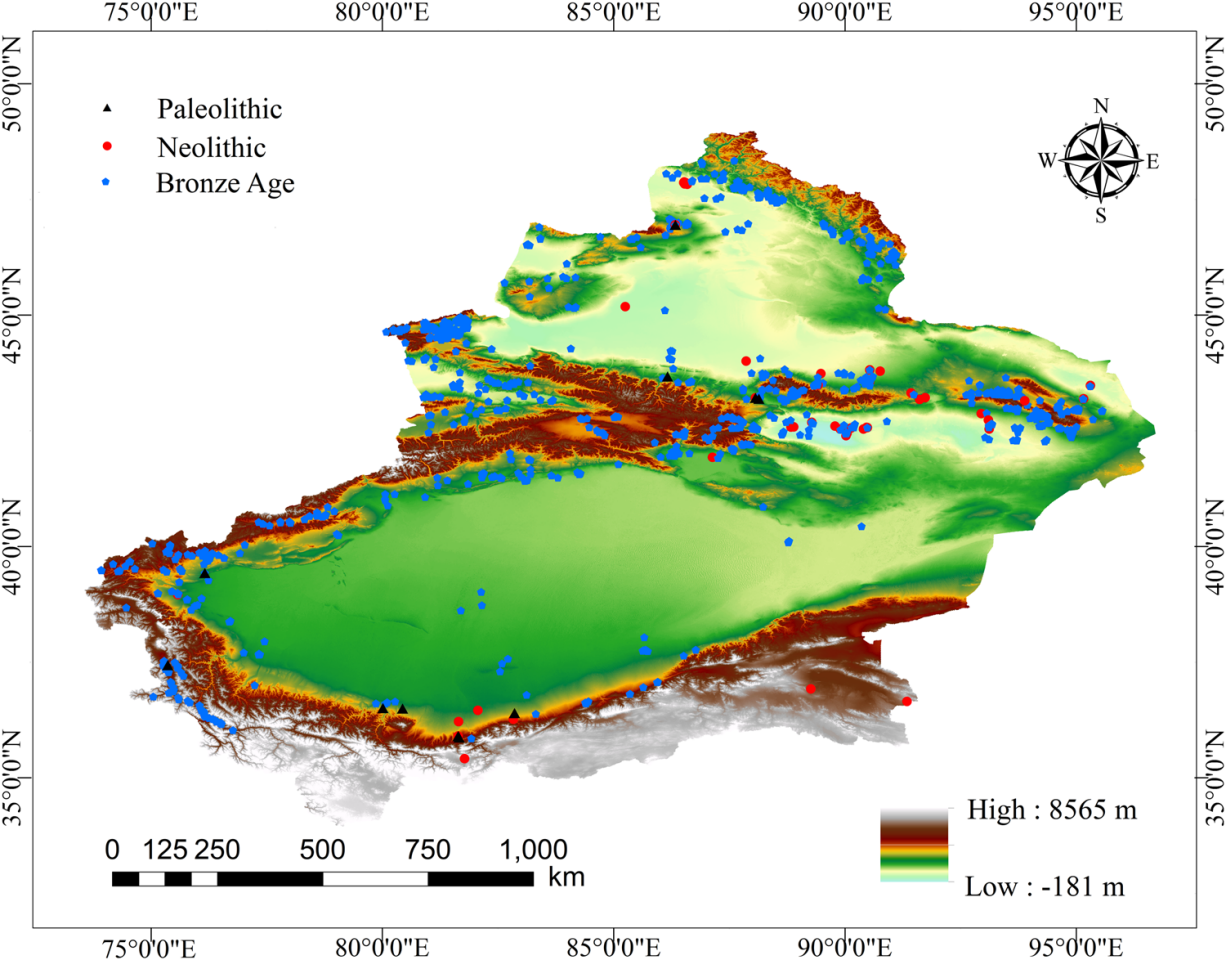
Distribution of cultural sites in different periods.

The site data used in this study is mainly based on the digitization of text data. The written materials come from the archaeological excavation results in Xinjiang for many years. They are supplemented by “The Atlas of Chinese Cultural Relics Xinjiang Volume” published by the State Administration of cultural relics^12^, “The Compilation of Cultural Relics and Archaeology in Xinjiang” published by the Xinjiang Institute of cultural relics and archaeology^18^, Huang Wenbi’s Collection of Archaeological Research^19,20^, The Statistical Yearbook of Chinese Archaeology issued by the Chinese Archaeological Society and the results of China’s third national cultural relics survey^21–40^. In this data, the sites with unknown ages are excluded; a total of 1655 sites are collected, including 12 sites of the Paleolithic, 52 sites of the Neolithic and 1591 sites of the Bronze age, as shown in Fig. 1. According to the position description in the written record, calibrate the site’s position in the satellite image, record its longitude and latitude information, and then proofread it through archaeological excavation report and sampling field investigation. The data set mainly includes immovable cultural relics, including a small number of inscriptions and rock paintings relocated for protection.

**Fig. 2:**
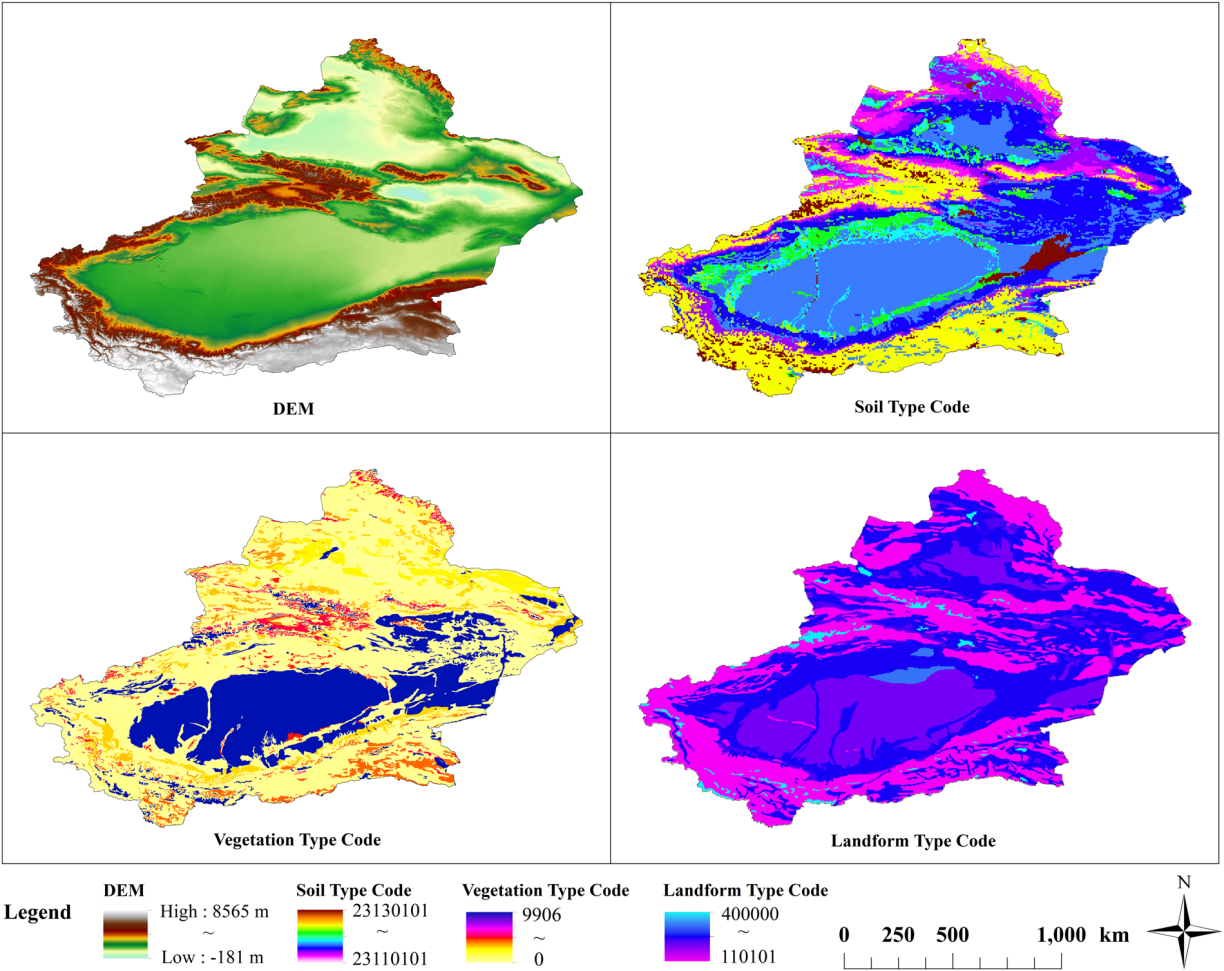
Basic data of geographic environment elements.

**Fig. 3:**
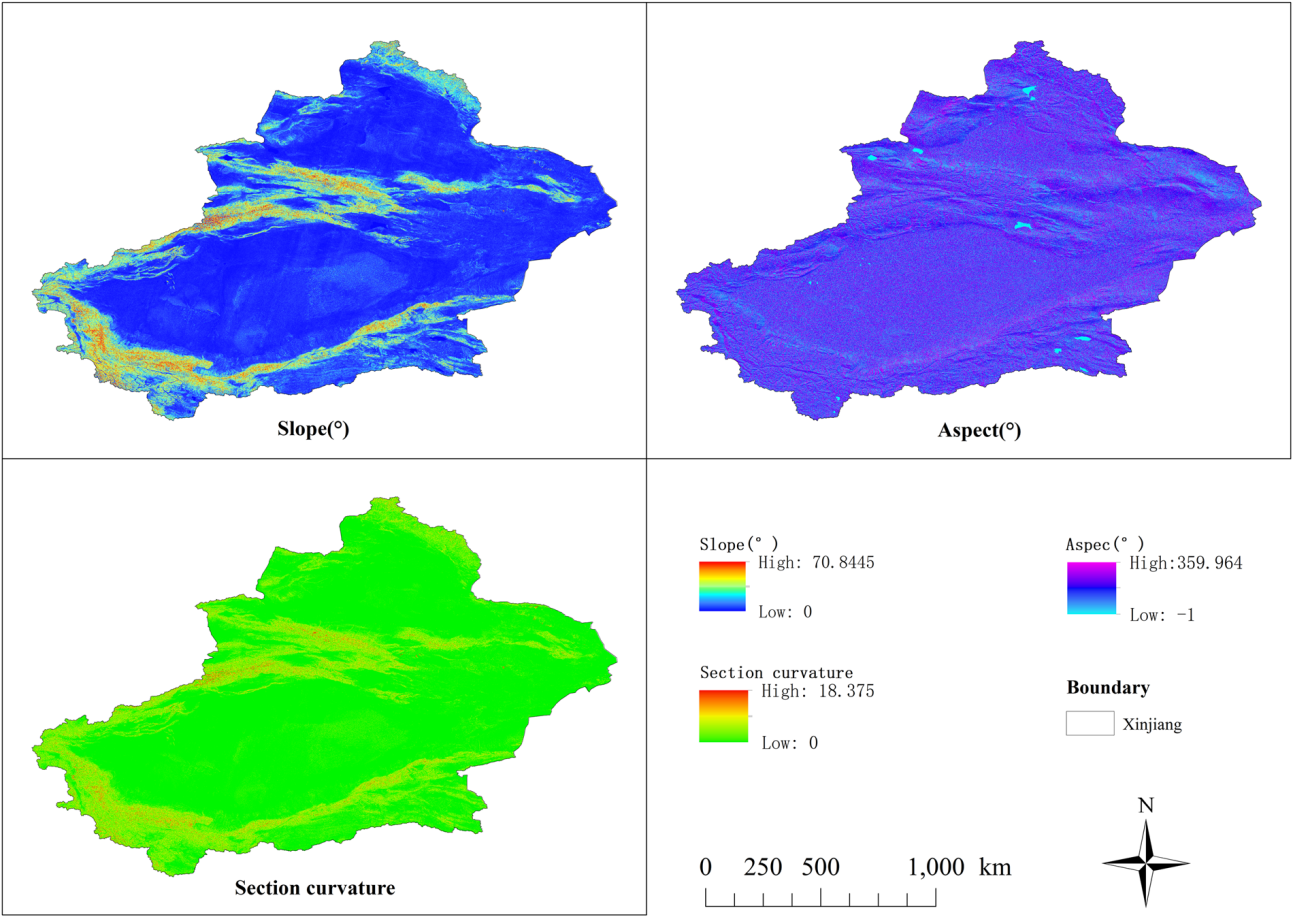
The extracted slope, aspect and profile curvature of the study area.

Based on the spatial location calibration, the GIS data of the site location is obtained through ArcGIS vectorization conversion. Then take the 30 m digital elevation, soil type data, vegetation type data, and geomorphic type data of China as the geographical environment background data (Fig. 2), the elevation, slope, profile curvature, slope direction, soil type, geomorphic type and other information of the production site are extracted and produced through ArcGIS 10.2. First, based on DEM data, the study used Spatial Analyst’s Slope and Aspect tools in ArcGIS to produce a slope, aspect, and profile curvature data, as shown in Fig. 3. Then use the Extract Values to Points tool to extract the raster information of all geographic background data to cultural sites Point data, output point data attributes and save it in CSV format to obtain a data set of site distribution and geographic environment information.

Digital elevation model (DEM) elevation data with a resolution of 30 m were obtained from the geospatial data cloud website (http://www.gcloud.cn), and 1:1 million-scale vegetation type spatial distribution data for China come from the Chinese Academy of Sciences Resource and Environmental Science Data Center (http://www.resdc.cn/Default.aspx)^41^. Chinese soil attribute data and landform type data come from the National Qinghai-Tibet Plateau Science Data Center (http://data.tpdc.ac.cn)^42,43^.

On the one hand, this study’s division of time stages comes from the archaeological age recorded in written materials. On the other hand, based on the simultaneous replacement of the cultural site excavation time and the cultural age of the Central Plains site, the cultural age is unified into Chinese prehistoric cultural archaeology Years to improve the universal applicability of the data. There are differences in the time of entering the Bronze Age across China, so the division of the Bronze Age in Xinjiang is still controversial. This article takes the time of Xinjiang Hami Linya cemetery as the beginning of the Bronze Age because the earliest bronzes in Xinjiang were found in this tomb^44^. At the same time, this article believes that the production and popularization of metal tools are two completely different types of productivity and civilization progress. Therefore, this article takes the time when the Iron Age was entered, when bronze tools were widely replaced, as the end of the Bronze Age, namely 4500a.Bp-2250a.Bp. The detailed era division is shown in Table 1.

**Table 1:** The time, Era sequence and Aspect division standard of site culture.

About the era division of the site			
Civilized age	Cultural age(Equivalent to)	Time (a, BP)	Chronological order
Paleolithic	Dingcun Culture	40,000–30,000	1
	Middle Pleistocene	30,000–26,000	2
	Late Pleistocene	29,000–27,000	3
	Late Pleistocene	19,000–17,000	4
	Pleistocene	16,000–11,000	5
Neolithic	Mesolithic stage	11,000–8,000	6
	Early Yangshao Culture	7,000–6,000	7
	Late Yangshao Culture	5,500–5,000	8
	Qijia Culture	4,500–4,000	9
	Longshan Culture	4,500–4,000	10
Bronze Age	Yanbulak Culture	4,000–3,000	11
	Xia Shang Culture	4,000–3,000	12
	Western Zhou Dynasty	3,100–2,700	13
	Eastern Zhou Dynasty	2,800–2,200	14
**Aspect Standard**			
**Positive degree of aspect**	**Aspect**	**Positive degree of aspect**	**Aspect**
338-22°	North	158–202°	South
23–67°	Northeast	203–247°	southwest
68–112°	East	248–292°	West
113–157°	Southeast	293–337°	Northwest

## Data Records

The raw data of this research has been released in the National Qinghai-Tibet Plateau Data Center (http://data.tpdc.ac.cn). 10.11888/HumanNat.tpdc.271910^45^. The website is http://data.tpdc.ac.cn/en/disallow/bb49a6da-bfd4-4355-9d0c-988eef793ee1/. The database is stored in the CSV format in The National Tibetan Plateau Data Center (TPDC) (http://data.tpdc.ac.cn/en/), and 16 pieces of information are collected for each site: (1) Chronological order; (2)) Longitude; (3) Latitude; (4) Site category; (5) Name; (6) Civilized age; (7) Cultural age(Equivalent to); (8) Area (m^2^); (9) Soil type code; (10) Landform Type Code; (11) Vegetation type code; (12) Altitude(m); (13) Slope(°); (14) Aspect; (15) Time(a, BP); (16) Section curvature. The “Chronological order” is based on the time of existence of each site, from Paleolithic to the Bronze Age, as shown in Table 1. “Longitude” and “Latitude” are the longitude and latitude of the cultural site and are the longitude where the cultural site sits, recorded in decimal format. Because some of the sites have been backfilled and buried on the ground and are not visible, most of the sites are confirmed by satellite image 2021, so the latitude and longitude are approximate values. “Site category” is the type of cultural relics defined by the classification method in the “Law of the People’s Republic of China on the Protection of Cultural Relics”. This article mainly includes ancient sites, ancient tombs, stone carvings, cave temples, and others, divided into 4 main types. “Name” represents the name of the site, based on the archaeological naming at the time the site was discovered, generally the village or natural location + type, such as Dushanzi (place name) tomb (type). “Civilized age” indicates the civilization stage of the site, namely the Paleolithic, Neolithic and Bronze Age. “Cultural age (Equivalent to)” is the chronology of Chinese history in which the cultural site is located. Some ancient humans and their remains use geological age. Due to the vague classification of archaeological types between the Paleolithic and Neolithic cultural sites in Xinjiang^12^, and the existence of regional cultural types are not clear, this article has made a one-to-one correspondence with the archaeological, cultural types of China’s Central Plains based on their chronological information^12,44,46^, the unified replacement is the cultural era of the Central Plains region. “Area (m^2^)” indicates the area of cultural sites. “Soil type code” represents the modern soil type code where the cultural site is located; see literature for the specific soil type. “Landform Type Code” represents the modern landform type code where the cultural site is located^42^; see literature for the corresponding landform type^43^. “Vegetation type code” represents the modern vegetation type code where the cultural site is located. For specific vegetation types, see literature^41^. “Altitude(m)” represents the altitude where the cultural site is located, in m. “Slope (°)” means the slope of the plane where the cultural site is located, and the unit is °. “Aspect” refers to the aspect of the plane where the cultural site is located. This article is divided into eight aspects: North, East, South, West, Northeast, Southeast, Northwest, and Southwest. The classification standards are shown in Table 1. “Time (a, BP)” means the age of the cultural site, the unit is a (year), BP is Before Present, and the specific period is shown in Table 1. Since the cultural type or historical dynasty age is adopted, and most sites lack absolute geological dating, the site’s age is an approximate value. “Section curvature” represents the section curvature of the plane where the cultural site is located, with 4 significant digits reserved.

## Technical Validation

The information on cultural sites recorded in this study comes from the written materials released by the State Administration of Cultural Heritage of China, the Xinjiang Institute of Cultural Relics and Archaeology and the Chinese Archaeological Society. The written materials are authoritative because these publishers belong to official Chinese institutions and top academic groups. At the same time, we combined the excavation reports of some scholars, news information and field verification to confirm the location information of the site. To extract geographic information, we use the highly recognizable geographic background data released by the current official scientific research institutions. There is no problem with the scientificity and standardization of the background data. However, different data were produced in different years, by different authors and institutions, lack of uniform standards for data recording, and different observation techniques. When we collect data information, we are inevitably affected by the potential impact of these problems, which limits the improvement of site positioning accuracy and data accuracy. In addition, due to the lack of detailed records of on-site excavations and interviews with many excavators, the cultural information of the site recorded by this data is limited, and the spatial-temporal accuracy of site information needs to be further improved. Because of the above two deficiencies, we are still collecting data and going to the field to verify as much as possible, to continuously update the data and improve its quality.The spatial position error should be between 0-1 km. However, from the perspective of data application, this data is more used for macro analysis on a large spatial scale than the discussion of microsite information requiring high-precision location information, and there is not such a large number of sites in the study of microsites. From this point of view, the spatial error of sites is understandable. For the analysis of micro-sites, this data can also provide high-precision spatial locations or a general range, which is also of great guiding significance for researchers, especially scholars who are not familiar with the distribution of Xinjiang cultural sites and their first field investigations.

## Usage Notes

From the current point of view, the application of this data is in the following aspects:

1. Research on the relationship between man and land in prehistoric times. Early humans existed and lived in Xinjiang. In the process of repeatedly adapting to climate change in Xinjiang, early human beings in Xinjiang transformed the landscape, built and retained a large number of sites. The collation and collection of these site information in this data can provide a data basis for the site selection characteristics of human settlements, the temporal and spatial evolution process and model research of prehistoric human adaptation and transformation of living environment in this area. It can also deduce and interpret the evolution of prehistoric human relations.
2. The proliferation process and spatial distribution characteristics of prehistoric humans. The origin, expansion process, and spatial distribution of human beings focus on environmental archaeology, anthropology, history, and geography. Firstly, this data records the age of each site, reflecting the sequence of site formation and the spatial process of human territory expansion. The spatial distribution characteristics of the site are the potential indicators of the spatial scope, density and scale of human activities. Therefore, the information mining of site data can reference the diffusion process and activity characteristics of prehistoric humans. Secondly, Xinjiang is a channel connecting China and Central Asia. Therefore, the in-depth combing of the cultural types of sites in this region can provide a reference for further understanding the East-West exchanges and the evolution of civilization in Eurasia.
3. Utilization and protection of the ruins. Prehistoric human sites are not only essential material materials for studying prehistoric humans. For modern society, they are also cultural heritage and important resources. Many large-scale sites are often built as museums or developed as tourism resources after excavation. Therefore, the site database can evaluate and develop tourism resources in the area. The site prediction based on the distribution characteristics of the existing sites can reveal the high probability areas of the site distribution, provide guidance for the critical areas of the site excavation, and can be applied to social and economic construction planning. In this way, the conflict between the excavation and protection of the ruins and the land for economic construction can be alleviated.

## Data Availability

No code was used in the creation of this data. Titles should avoid the use of acronyms and abbreviations where possible. Colons and parentheses are not permitted.
